# Isolation, Molecular Identification, and Mycotoxin Production of *Aspergillus* Species Isolated from the Rhizosphere of Sugarcane in the South of Iran

**DOI:** 10.3390/toxins12020122

**Published:** 2020-02-14

**Authors:** Maryam Tavakol Noorabadi, Valiollah Babaeizad, Rasoul Zare, Bita Asgari, Miriam Haidukowski, Filomena Epifani, Gaetano Stea, Antonio Moretti, Antonio Francesco Logrieco, Antonia Susca

**Affiliations:** 1 Department of Plant Protection, Sari Agricultural Sciences and Natural Resources University, 48181 68984 Sari, Iran; maryam.tavakol65@yahoo.com (M.T.N.); babaeizad@yahoo.com (V.B.); 2 Iranian Research Institute of Plant Protection, Agricultural Research, Education and Extension Organization (AREEO), 19858 13111 Tehran, Iran; simplicillium@gmail.com (R.Z.); bita_asgari@yahoo.com (B.A.); 3 Institute of Sciences of Food Production, National Research Council of Italy, 70126 Bari, Italy; miriam.haidukowski@ispa.cnr.it (M.H.); filomena.epifani@ispa.cnr.it (F.E.); gaetano.stea@ispa.cnr.it (G.T.); antonio.logrieco@ispa.cnr.it (A.F.L.)

**Keywords:** Beta-tubulin, calmodulin, fumonisins, ochratoxin A, patulin, mycotoxin, sugarcane, *Aspergillus*

## Abstract

Knowledge of the genetic diversity detected among fungal species belonging to the genus *Aspergillus* is of key importance for explaining their important ecological role in the environment and agriculture. The current study aimed to identify *Aspergillus* species occurring in the rhizosphere of sugarcane in the South of Iran, and to investigate their mycotoxin profiles. One-hundred and twenty-five *Aspergillus* strains were isolated from the soil of eight major sugarcane-producing sites, and were molecularly identified using sequences of partial β-tubulin (*benA*) and partial calmodulin (*CaM*) genes. Our molecular and phylogenetic results showed that around 70% of strains belonged to the *Aspergillus* section *Nigri*, and around 25% of species belonged to the *Aspergillus* section *Terrei*. Species belonging to both sections are able to produce different mycotoxins. The production of mycotoxins was measured for each species, according to their known mycotoxin profile: patulin (PAT) and sterigmatocystin (STG) for *Aspergillus terreus*; ochratoxin A (OTA) and fumonisins for *Aspergillus* *welwitschiae*; and OTA alone for *Aspergillus tubingensis*. The data showed that the production of OTA was detected in only 4 out of 10 strains of *A. welwitschiae*, while none of the *A. tubingensis* strains analyzed produced the mycotoxin. Fumonisins were produced by 8 out of 10 strains of *A. welwitschiae*. Finally, none of the 23 strains of *A. terreus* produced STG, while 13 of them produced PAT. The occurrence of such mycotoxigenic plant pathogens among the fungal community occurring in soil of sugarcane fields may represent a significant source of inoculum for the possible colonization of sugarcane plants, since the early stages of plant growth, due to the mycotoxin production capability, could have worrisome implications in terms of both the safety and loss of products at harvest.

## 1. Introduction

Sugarcane (*Saccharum officinarum* L., family *Poaceae*) is one of the most important agricultural crop plants in Iran, where it is cultivated in the southern regions, with about 68,350 hectares and an annual production of about 5,685,000 tons [1]. The high importance of sugarcane cultivation is due to its multiple exploitations: production of ethanol coupled with the search for cleaner energy sources, as well as the electrical and/or thermal energy [2] production of citric acid, as the source of key players of fermentation, *Aspergillus niger* and sugarcane bagasse [3]; production of antibiotics [4], organic acids [5,6] medicines, or enzymes [4,7,8,9]; and supply of important green material for industries that produce sugar and by-products [10,11]. The latter two uses are related to human and animal food consumption, so much attention must be devoted to the safety of sugarcane products, such as possible mycotoxin contamination.

Microbial communities occurring in the soil of sugarcane crops are of biological importance to sugarcane because the crop is produced by successive stalk harvesting from initial stalk-cutting plantations, which are annually left in the soil to produce the next plant generation. Therefore, these stalks represent the major source of inoculum of plant contamination [12]. Indeed, over 90% of all microorganisms present in roots, stalks, and leaves are also present in bulk soil samples [13], suggesting that the microbial diversity present in the bulk soil is also represented in the plant organs at early stages of plant development. Mycobiota, both in the field and post-harvest, under specific temperatures and humidity, can use the sugar as a source of energy for their growth. Furthermore, they can produce secondary metabolites, such as mycotoxins, which can both cause processing problems in the mill and refinery, and be toxic to animals and humans, e.g., through mycotoxin production [11]. *Aspergillus* species are among the most important mycotoxigenic fungi. In the genus, there are both species useful for industrial production and species that have harmful effects [14], with strong and varied biological activities ranging from moderate allergies to severe asthma and cancer [15].

The black aspergilli are an important group of fungi that can colonize food, feed at various stages, and cause the bio-deterioration of other materials. One of the species in the section *Nigri* that is extensively used in biotechnological processes is *A. niger*, whose fermentation process is “generally recognized as safe” by the Food and Drug Administration (FDA) in certain industrial conditions [16,17]. However, some species of the section *Nigri* have been reported as producers of mycotoxins, such as ochratoxin (OTA) and fumonisin (FB2) [18], which can thus affect the safety of sugarcane and its related biotechnological products. The section *Terrei* includes economically important species, isolated from different sources, that are very important in the fermentation industry [19,20], such as *Aspergillus terreus*, used to produce different enzymes and organic acids [21]. This species can produce a wide range of metabolites, some of which, like patulin, have important health effects and have been shown to be carcinogenic [22,23].

Because of the increased production of sugarcane in Iran and the level of by-products generated during processing, there is a need to monitor the presence of toxigenic fungal species occurring in soil that have the potential to colonize plants. In this context, we focused on *Aspergillus* species associated with sugarcane in the South of Iran, and investigated the related biodiversity, through DNA sequencing and possible mycotoxin production in vitro, in order to establish appropriate practices for crop management aimed at reducing the risk of contamination of sugarcane plants.

## 2. Results

### 2.1. Species Identification

**Figure 1:**
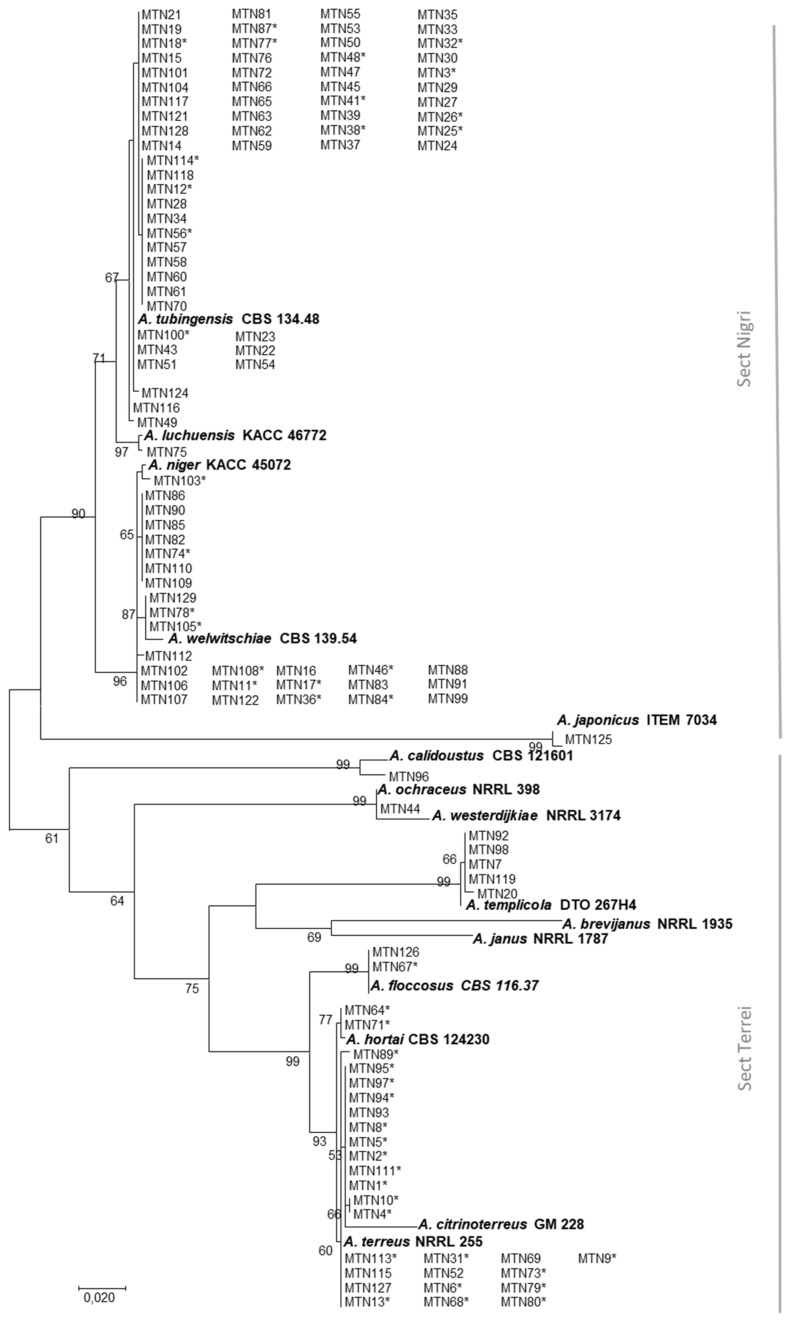
Phylogram generated for 125 strains with maximum likelihood analysis, based on a combined sequence dataset of β-tubulin and calmodulin (1556 bp). Bootstrap values > 50% (1000 replicates) are shown above or below the nodes. The scale bar indicates nucleotide substitution in ML analysis. The asterisk (*) indicates strains tested for mycotoxin production.

To identify *Aspergillus* species occurring in the sugarcane rhizosphere in the South of Iran, and to investigate their mycotoxin profiles, 64 samples were collected from eight major sugarcane-producing sites (Table 1). In total, 125 strains of *Aspergillus* were isolated and, based on morphological characteristics, were primarily divided into five sections, including *Circumdati*, *Flavipedes*, *Nigri*, *Terrei*, and *Usti*. However, the majority of strains were included in sections *Nigri* and *Terrei*. For precise species identification, all isolates were subjected to a DNA-based analysis of partial β-tubulin (benA) and partial calmodulin (CaM) genes. The concatenated sequences of benA and CaM, generated in this study, were aligned against the sequences of 13 reference *Aspergillus* species available at GenBank. Based on our phylogenetic analysis (Figure 1), nine species could be identified: *Aspergillus calidoustus* (1 strain), *Aspergillus japonicus* (1), *Aspergillus luchuensis* (1), *A. niger* (2), *Aspergillus ochraceus* (1), *Aspergillus templicola* (5), *A. terreus* (29), *Aspergillus tubingensis* (60), and *Aspergillus welwitschiae* (25).

**Figure 2:**
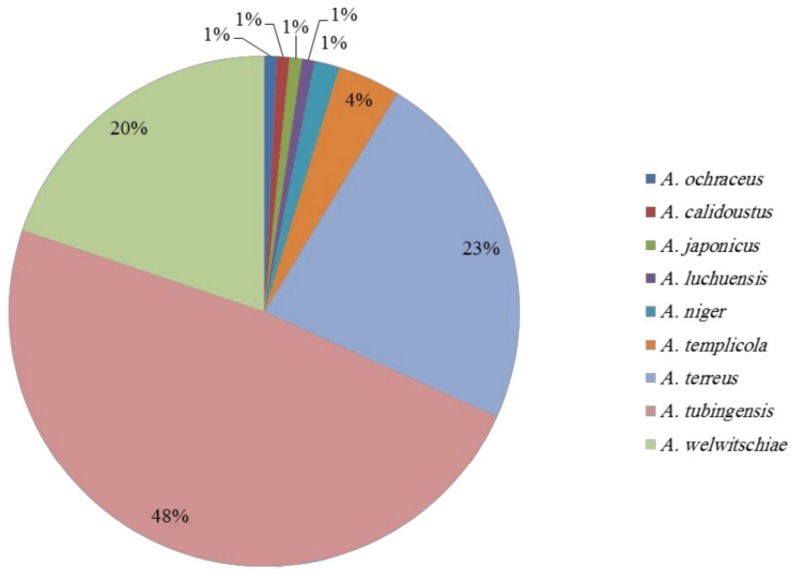
Distribution of *Aspergillus* species isolated from the sugarcane rhizosphere in the South of Iran.

Among the 125 *Aspergillus* strains, *A. tubingensis* occurred at a rate of 48%, *A. welwitschiae* at 20%, and *A. terreus* at 23%, together representing 89% of the *Aspergillus* population (Figure 2). In most of the Iranian regions, the percentage of *Aspergillus* section *Nigri* strains was higher than that of *Aspergillus* section *Terrei*. The highest occurrence of *A. tubingensis* was observed in Amir Kabir, Imam Khomeini, Debal Khazaei, Mirza K. Khan, and Salman Farsi, with a 75%, 50%, 73%, 82%, and 81% occurrence, respectively. In only one case (from Dehkhoda), *A. terreus* displayed the highest occurrence (80%). On the other hand, *A. welwitschiae* was the most frequent species in Karun and Haft Tappeh regions (42% and 30%, respectively).

### 2.2. Mycotoxin Production

Mycotoxin production of a subset of strains, representative of the different sites of isolation, was tested for potential toxigenic species: *A. welwitschiae* (10 strains), *A. tubingensis* (15 strains), and *A. terreus* (23 strains) (see Table 2).

No production of ochratoxin A (OTA) was observed among the 10 tested strains of *A. welwitschiae*, when grown on CY20S; however, on YES medium, four out of the 10 strains produced OTA, in the range of 32 and 142 µg/g. None of the 15 *A. tubingensis* strains, grown on both CY20S and YES media, were able to produce OTA. Fumonisin B2 (FB2) was produced by eight out of 10 *A. welwitschiae* strains tested (MTN108, MTN46, MTN11, MTN17, MTN105, MTN78, MTN103, and MTN36) in the range of 0.1 and 4.4 µg/g, when grown on CY20S. Furthermore, 13 out of the 23 tested strains of *A. terreus*, on YES, were able to produce patulin (PAT) in the range 0.2 and 523.56 µg/g. On the other hand, no production of PAT was recorded by the same strains when grown on CYA. Finally, sterigmatocystin (STG) was not produced by any strain of *A. terreus*, on both YES and CY20S (Table 2).

## 3. Discussion

Agricultural crops grow in soils, which usually host enormous types and numbers of micro-organisms, that, from one side, are responsible for soil health by nutrient cycling, while from the other side, can opportunistically infect the plants [24,25,26]. Among micro-organisms, fungal species of the genus *Aspergillus* are soilborne micro-organisms, and their predominance in rhizospheric and non-rhizospheric soils has been extensively studied [27]. In this study, we focused our analyses on the isolation and identification of *Aspergillus* species, since they are well-known mycotoxin-producers and their occurrence in sugarcane soils can be the cause of a subsequent contamination of sugarcane plants that grow in such contaminated soils [28].

**Figure 3:**
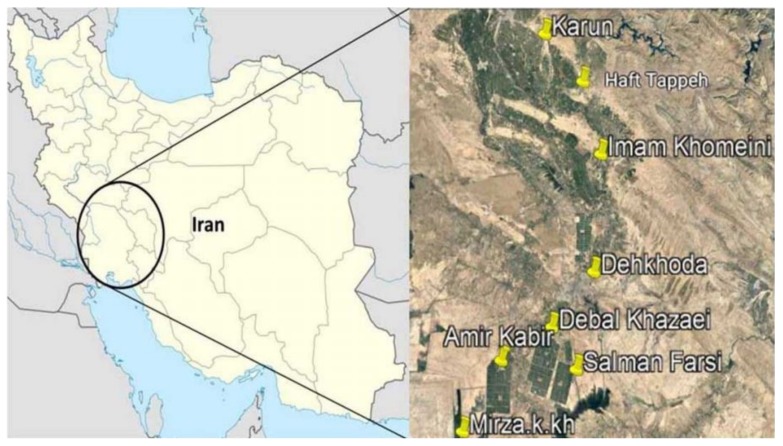
The geographical origins of *Aspergillus* strains isolated from the sugarcane rhizosphere in the South of Iran. Pins indicate the areas where the rhizosphere samples were collected.

The occurrence of *Aspergillus* species was evaluated in the rhizosphere of eight sugarcane sites in the South of Iran (see Figure 3 and Table 3), where the sugarcane is the main agro-food product. The number of *Aspergillus* strains isolated in this study varied from region to region: Imam Khomeini and Haft Tappeh sites showed the highest and lowest number of isolates, respectively. These differences in the distribution of strains among the regions might be related to significant specific environmental and biological features of each site, such as the microbiome profile [29,30], fungal antagonist occurrence [31], and soil moisture and temperature [32]. However, inadequate knowledge on the sites sampled prevents us from drawing further conclusions.

The presence of toxigenic *Aspergillus* species in the soil associated with sugarcane indicates that a contamination of sugarcane plants in the field and its by-products post-harvest can occur. The heat treatment with which sugarcane juice is processed to obtain molasses and non-refined sugar, leads to the removal of fungal contaminants at this stage. However, since we proved a high presence of toxigenic *Aspergillus* species in the rhizosphere of sugarcane plants, the contamination of the early stages of plant development could be high. Therefore, in the field, environmental conditions suitable for mycotoxin production in planta by the *Aspergillus* species can easily occur and also lead to the contamination of final products. As a consequence, this contamination can cause a high toxigenic risk for consumers, but can also interfere with sugarcane industrial processing. 

Our molecular and phylogenetic results showed that around 70% of strains belong to the *Aspergillus* section *Nigri*, and around 25% of species belong to the *Aspergillus* section *Terrei*. Although no morphological differences were observed among strains assigned to *A. terreus*, a phylogenetic tree divided them into two clades. Interestingly, MTN64 and MTN71, which were morphologically identified as *A. terreus*, were closely related to *A. hortai* (CBS124230). A correct identification of *Aspergillus* species is a key aspect, since many species produce mycotoxins and each species has its own mycotoxin profile. Therefore, a precise risk assessment is strongly linked to the use of advanced diagnostic tools.

As reported above, the species identified in this survey belonged to two main *Aspergillus* sections—*Terrei* and *Nigri*—characterized by distinct mycotoxin profiles: PAT and STG for section *Terrei* and OTA and FB2 for section *Nigri*. The in vitro mycotoxin production by different species tested was analyzed according to their known mycotoxin profile: PAT and STG for *A. terreus* and OTA and FB2 for *A. welwitschiae*. However, the production of OTA was also tested for *A. tubingensis* because its ability to produce mycotoxin has been inconsistently reported. Susca et al. [33] demonstrated the lack of some genes in the OTA cluster, most likely related to their inability to produce OTA, while Medina et al. [34] reported OTA production for few strains of *A. tubingensis*. However, our data confirmed the inability of *A. tubingensis* to produce OTA, since none of the strains tested could produce any trace of the mycotoxin. The fact that this species is the most frequently detected in our study is comforting since it reduces the risk related to the occurrence of mycotoxigenic *Aspergillus* species.

On the contrary, the presence of *A. welwitschiae* strains that produce both OTA and FB2 is worrisome, since both of these mycotoxins are considered by the International Agency for Cancer Research (IARC) group 2b, meaning potentially carcinogenic. Only a minority of strains analyzed were able to produce only OTA, while the majority of *A. welwitschiae* strains (80%) also produced FB2, in agreement with previous reports [33,35]. Some authors have demonstrated that OTA production in *A. niger* is affected by temperature [36], while in *A. welwitschiae*, it is affected by water activity and the medium [37]. Our data confirm the high influence of media in the in vitro production of OTA by *A. welwitschiae*. No strain produced the mycotoxin on CY20S, while 40% of strains produced it if grown on YES. In addition, since the temperature was proved to affect OTA production, environmental and geographical variations clearly influence the potential of OTA production by *A. welwitschiae* in the field of a given area.

To the best of our knowledge, in Iran, there is currently a lack of information on the mycotoxin contamination of sugarcane and related by-products. Here, we reported the potential contamination by FB2, OTA, and PAT of sugarcane plants. Additionally, our data suggest the need for further investigations for developing a better understanding of fungal contamination and related mycotoxins in planta, in order for proper management protocols to be adopted and to minimize the risk of contamination of this prestigious food. Finally, the high variability of the *Aspergillus* species profile in the soils of different regions, the high influence of temperature on their ability to colonize soils, and the rapidity with which the population structure of the genus *Aspergillus* can change in the soil, together suggest that, by the time a study like this one is published, the data presented in it are historical rather than a profile of an existing *Aspergillus* population. However, genetic diversity and flux in populations of the genus *Aspergillus* may allow us, in Blake’s words, ‘to see the world in a grain of sand, and eternity in an hour’.

## 4. Conclusions

Our study showed the presence of toxigenic *Aspergillus* species in the rhizosphere of sugarcane. Such occurrence could represent a toxigenic risk for the crop culture in the following year, due to the usual practices of harvesting in Khuzestan province. By testing the in vitro toxin production of fungal species isolated, this study showed their capability of producing OTA, PAT, and FB2, and therefore, this might be the cause of possible contamination of sugarcane plants. These data demonstrate the need for further investigations aimed at assessing the possible risk for human and animal health, due to the consumption of sugarcane products contaminated by mycotoxins.

## 5. Materials and Methods

### 5.1. Samples

Sugarcane fields were sampled from different regions in the South of Iran, depending on the history of cultivation. The sampling was conducted in different stages of sugarcane development, preferably in cold seasons (autumn and winter), with at least 20 well-distributed samples per field, including sugarcane root with the surrounding rhizosphere [38]. Samples were placed in suitable paper envelopes and transferred to the laboratory. After transferring samples to the laboratory, the rhizosphere soil around the root was carefully separated from the root. Samples were divided into soil and root subunits. Soil samples were kept in a ventilated condition and preferably at a temperature of 15–20 °C for 12 to 24 h (depending on the moisture content of the soil) and then transferred to the refrigerator (2–5 °C) for further examinations [39].

### 5.2. Fungal Isolation and Morphological Identification

The soil dilution plating technique modified by Johnson et al. [40] and Warcup [41] was used to isolate *Aspergillus* species from the soil. Soil samples were crushed by a 2 mm sieve. About 10 gram of the crushed soil was poured into a graduated cylinder. One hundred milliliters of sterile water was added to the cylinder. The suspension was transferred to the 250 mL Erlenmeyer flask and mixed for 30 minutes. One milliliter of suspension was transferred to test tubes containing 9 mL of 0.12% water agar medium. The 1:100, 1:1000, and 1:5000 dilutions were prepared. One milliliter of 1:1000 and 1:5000 dilutions was transferred to Petri dishes and then 10–12 mL of PCA (Potato Carrot Agar) medium [42] containing melted and cooled 1.5% agar and 1% oxgall was added. In total, 200 ppm of penicillin and streptomycin antibiotics were also added to avoid bacterial contamination. The plates were moved gently so that the suspension became uniform. For each dilution, three plates were considered. The plates were incubated in the dark at 17 °C for 10 days. Pure cultures were obtained by single spore isolations. Some representative strains—48 out of the 125 included in the study—were deposited in ITEM (Agri-Food Toxigenic Fungi Culture Collection, ISPA-CNR Bari, Italy, http://server.ispa.cnr.it/ITEM/Collection/) and IRAN (Iranian Fungal Culture Collection, Iranian Research Institute of Plant Protection, Tehran, Iran, http://gcm.wfcc.info/cc/iran).

For morphological characterization, pure cultures were grown on Malt Extract Agar (MEA), and were incubated at 25 °C for 7 days. Macroscopic traits, such as the colony appearance, color, pigmentation, and growth rate, were recorded according to standard protocols [27,43,44,45].

### 5.3. Molecular Identification

For molecular identification, isolates were grown on Potato Dextrose Agar (PDA). Direct PCR from fungal mycelia was done using Phire Plant Direct PCR Master Mix (F-160L, Thermo Fisher Scientific, Waltham, USA). For direct PCR, a small piece of growing mycelium from a 3–4-day-old colony was swiped with a sterile pipette tip and re-suspended in 20 μL of 1× Phire Plant Direct PCR Master Mix prior to PCR. Mycelia were crushed with a 100 μL pipette tip and vortexed briefly, and then collected down at the tube bottom with a spin in centrifuge. The supernatant (1 μL) was used as a template for a 20 μL PCR reaction. All amplifications were performed according to standard Direct PCR protocols (Table 1 and Table 2) by using a GeneAmp 9700 thermalcycler (Applied Biosystems, Foster City, CA, USA). Partial β-tubulin (*benA*) and partial calmodulin (*CaM*) genes were amplified using primers described in the literature: primers BT2a and BT2b, and CL1 and CL2A [46,47]. After amplification, amplicons were purified with the enzymatic mixture EXO/SAP (Exonuclease I, E. coli/Shrimp Alkaline Phosphatase) and used as a template for bidirectional DNA sequencing. Sequencing was performed with the BigDye v3.1 terminator kit (Applied Biosystems, Foster City, CA, USA), following the manufacturer’s instruction, and analyzed on an ABI 3730 XL Genetic Analyzer (Applied Biosystems, Foster City, CA, USA). Alignment of the two strands was performed using the software package BioNumerics 5.1 (Applied Maths, Sint-Martens-Latem, Belgium)), with manual adjustments, where necessary. Sequences of *benA* and *CaM* genes, generated in this study, were deposited in GenBank with the following accession numbers: from LR693748 to LR693997.

### 5.4. Sequence Data Analysis

To obtain a previous species identification in order to select reference sequences for *Aspergillus* species to be used in the phylogenetic analysis, sequences of *benA* and *CaM* were searched for on the GenBank database using the Basic Local Alignment Tool (BLASTN, NCBI BLAST website). The sequences of 125 strains were used to perform phylogenetic analysis. A set of 16 reference sequences for *Aspergillus* species, identified by BAST analysis, were downloaded from GenBank and used for phylogenetic analysis. All sequences were aligned using the MUSCLE algorithm [48] with MEGA7 software ver. 7.0.14 [49]. The evolutionary history was inferred by using the Maximum Likelihood method based on the Tamura–Nei model in MEGA7 software [50]. To evaluate the support for inferred topologies, the percentage of trees in which the associated taxa clustered together was calculated by bootstrapping with 1000 replicates [51]. The percentage of trees in which the associated taxa clustered together is shown next to the branches. Initial trees for the heuristic search were obtained automatically by applying Neighbor-Join and Bio NJ algorithms to a matrix of pairwise distances estimated using the Maximum Composite Likelihood (MCL) approach, and then selecting the topology with a superior log likelihood value. The tree was drawn to scale, with branch lengths measured in the number of substitutions per site. The analysis involved 138 nucleotide sequences. Codon positions included were 1st + 2nd + 3rd + Noncoding. All positions containing gaps and missing data were eliminated.

The phylogenetic approach was based on *benA* and *CaM* concatenated sequences, inferred from 125 strains of *Aspergillus* examined in this study compared to 13 reference *Aspergillus* species available at GenBank: *A. niger* (KACC 45072, AY585542, and JX500080), *A. tubingensis* (CBS134.48, AY820007, and AJ964876), *A. japonicus* (ITEM7034, AY585542, and AJ964875), *A. welwitschiae* (CBS139.54, FJ629291, and KC480196), *A. calidoustus* (CBS121601, EF591730, and HE616559), *A. citrinoterreus* (GM228, LN680657, and LN680685), *A. floccosus* (CBS116.37, FJ491714, and FJ531219), *A. hortai* (CBS124230, FJ491706, and KP987054), *A. luchuensis* (KACC46772, JX500062, and JX500071), *A. ochraceus* (NRRL398, EF661322, and EF661381), *A. templicola* (DTO267H4, KJ775087, and KJ775371) *A. terreus* (NRRL255, EF669519, and EF669544), and *A. westerdijkiae* (NRRL 3174, EF661329, and EF661360).

### 5.5. Mycotoxin Analyses

Methanol, acetonitrile (both for HPLC purposes), and glacial acetic acid were purchased from VWR International Srl (Milan, Italy). Ultrapure water was produced by a Millipore Milli-Q system (Millipore, Bedford, MA, USA). Ochratoxin A (OTA), patulin (PAT), and sterigmatocystin (STG) (purity > 99%) were produced by Sigma-Aldrich (Milan, Italy). Fumonisin B2 (FB2) was purchased from Biopure (Romer Labs Diagnostic GmbH, Getzersdorf, Austria). An RC 0.2 μm filter (regenerated cellulose membranes) was obtained from Grace (Grace Davison Discovery Science, Columbia, DC, USA). 

OTA stock solution was prepared by dissolving the solid commercial toxin in methanol (1 mg/mL). The exact concentration of OTA was determined according to the Association of Official Analytical Chemists (AOAC) Official Method 2001.01 [52]. Appropriate aliquots of the stock solution were brought to dryness and reconstituted with acetonitrile/water/acetic acid (99:99:2, v/v/v) to obtain standard solutions of OTA in the range 0.05–0.10 µg/mL. Fumonisin calibration solutions were prepared by diluting the samples with acetonitrile/water (1:1, v/v), to obtain solutions with a concentration in a range of 0.01–1.00 µg/mL for FB1 and FB2. Standard solutions were stored at −20 °C and warmed to room temperature prior to use.

PAT stock solution was prepared by dissolving the solid commercial toxin in ethanol (10 µg/mL). The exact concentration of standard solution was determined as reported by the European Committee for Standardization [53]. Aliquots of the stock solution were transferred to 4 mL amber glass vials and evaporated to dryness under a stream of nitrogen at 50 °C. The residue was dissolved in water/acetonitrile (85:15, v/v) to obtain a desired final concentration of 80 to 800 ng/mL.

Mycotoxin stock solution of STG (1 mg/mL) in acetonitrile was transferred to 4 mL amber silanized glass vials and evaporated to dryness under a stream of nitrogen at 50 °C. The residue was dissolved in water/methanol (60:40, v/v) to obtain calibrant standard solutions from 0.5 to 5.0 µg/mL. Standard solutions were stored at -20 °C and warmed to room temperature before use. Strains were grown for 14 days on YES (OTA, PAT, and STG), CYA (PAT and STG), and CY20S (FB2) media. One gram of agar was extracted with 5 mL of extraction solution on an orbital shaker for 60 minutes: OTA and PAT acetonitrile/methanol/water (90: 90: 80, v/v/v), FB2 methanol/water (70:30, v/v), and STG methanol/water (80: 20, v/v). Two milliliters of extracts were evaporated to dryness under a stream of nitrogen at 50 °C.

The quantification of mycotoxins was determined differently. OTA residue was dissolved with 1 mL of acetonitrile/water/glacial acetic acid (99:99:2, v/v/v) and filtered using RC through a 0.20 µm regenerated cellulose filter and determined by HPLC/FLD. OTA quantification was performed according to Susca et al. [35]. The quantification limit (LOQ) was 0.05 µg/g based on a signal to noise ratio of 10:1. FB2 residue was dissolved with 1 mL of the extract acetonitrile/water (30:70, v/v), filtered using RC 0.20 µm filters (Phenomenex, Torrance, CA, USA), and determined by HPLC/FLD previously derivatized with o-phtaldialdehyde (OPA). FB2 quantification was performed according to Susca et al. [35]. The LOQ of the method was 0.1 µg/g, based on a signal to noise ratio of 10:1.

PAT residue was dissolved with 1 mL of water/acetonitrile (85:15, v/v) and filtered using RC through a 0.20 µm regenerated cellulose filter and determined by HPLC/DAD. A total of 50 μL of extract was injected into HPLC apparatus Agilent 1260 Series. The analytical column was a Luna-C18 (4.6 × 150 mm, 5 µm) (Phenomenex, Torrance, CA, USA) preceded by C18 guard column (4 × 3 mm, Phenomenex, Torrance, CA, USA). The temperature of the column was maintained at 30 °C. The mobile phase was a mixture of water as solvent A and acetonitrile as solvent B, eluted at a flow rate of 1 mL/min. A gradient elution was performed as follows: 5% B solvent that was linearly increased by 100% in 15 min. In these analytical conditions, the retention time of PAT was about 5 min. The diode array detector (DAD) was set at a wavelength of 276 nm. The LOQ was 0.015 µg/g, based on a signal to noise ratio of 10:1 [54].

STG residue was dissolved with 1 mL of water/acetonitrile (75:25, v/v) and filtered through RC 0.20 µm. A total of 50 µL of extract was injected into HPLC apparatus Agilent 1260 Series. The analysis of toxins was performed using the following analytical method [55]. The analytical column was a Luna-C18 (4.6 × 150 mm, 5 µm) (Phenomenex, Torrance, CA, USA) preceded by C18 guard column (4 × 3 mm, Phenomenex, Torrance, CA, USA). The mobile phase consisted of a mixture of acetonitrile/water (40:60, v/v) at a flow rate of 0.8 mL/min. The array detector (DAD) was set at wavelengths of 248 and 328 nm. The temperature of the column was maintained at 30 °C. In this analytical condition, the retention time of ST was about 6.5 min. ST was quantified by measuring peak areas at the retention time of ST standard solutions and comparing these areas with the relevant calibration curve. The LOQ was 0.012 µg/g, based on a signal to noise ratio of 10:1.6. 

## Figures and Tables

**Table 1:** Occurrence of *Aspergillus* species isolated from the sugarcane rhizosphere in the South of Iran *.

Sampling Sites	No. of Sugarcane Rhizosphere Samples	No. of *Aspergillus* Isolates	*Aspergillus* spp. Occurrence (%)			
			*A. terreus*	*A. tubingensis*	*A. welwitschiae*	Others
Amir Kabir	8	8	- -	75	12.5	12.5
Imam Khomeini	8	4	25	50	25	- -
Debal Khazaei	8	11	- -	73	18	9
Dehkhoda	8	10	80	10	- -	10
Karun	8	23	25	29	42	4
Mirza K. Khan	8	11	9	82	9	- -
Salman Farsi	8	26	19	81	- -	- -
Haft Tappeh	8	32	21	24	30	24
Total	64	125	23	48	20	9

**Table 2:** Fumonisin (FB2), ochratoxin A (OTA), patulin (PAT), and sterigmatocystin (STG) production by representative strains of toxigenic species: *A. welwitschiae*, *A. tubingensis*, and *A. terreus*.

ITEM Collection Acc. Number	IRAN Collection Acc. Number	Isolate ID	Species	Mycotoxin Production (μg/g)		
				FB2	OTA	PAT *
18460	3629	MTN11	*A. welwitschiae*	1.0	106	n.t.
18461	3533	MTN17	*A. welwitschiae*	1.0	32	n.t.
18462	3550	MTN36	*A. welwitschiae*	<LOD	<LOD	n.t.
18464	3632	MTN46	*A. welwitschiae*	1.0	<LOD	n.t.
18465	3583	MTN74	*A. welwitschiae*	<LOD	<LOD	n.t.
18466	3587	MTN78	*A. welwitschiae*	0.1	<LOD	n.t.
18467	3606	MTN100	*A. tubingensis*	n.t.	<LOD	n.t.
18468	3608	MTN103	*A. welwitschiae*	4.4	<LOD	n.t.
18469	3609	MTN105	*A. welwitschiae*	0.2	49	n.t.
18470	3612	MTN108	*A. welwitschiae*	2.2	142	n.t.
18471	3521	MTN3	*A. tubingensis*	n.t.	<LOD	n.t.
18472	3528	MTN12	*A. tubingensis*	n.t.	<LOD	n.t.
18473	3530	MTN14	*A. tubingensis*	n.t.	<LOD	n.t.
18474	3534	MTN18	*A. tubingensis*	n.t.	<LOD	n.t.
18475	3541	MTN25	*A. tubingensis*	n.t.	<LOD	n.t.
18476	3542	MTN26	*A. tubingensis*	n.t.	<LOD	n.t.
18477	3547	MTN32	*A. tubingensis*	n.t.	<LOD	n.t.
18478	3552	MTN38	*A. tubingensis*	n.t.	<LOD	n.t.
18479	3554	MTN41	*A. tubingensis*	n.t.	<LOD	n.t.
18480	3559	MTN48	*A. tubingensis*	n.t.	<LOD	n.t.
18481	3567	MTN56	*A. tubingensis*	n.t.	<LOD	n.t.
18482	3586	MTN77	*A. tubingensis*	n.t.	<LOD	n.t.
18483	3595	MTN87	*A. tubingensis*	<LOD	<LOD	<LOD
18484	3616	MTN114	*A. tubingensis*	<LOD	<LOD	<LOD
18485	3520	MTN1	*A. terreus*	n.t.	n.t.	523.56
18486	3627	MTN2	*A. terreus*	n.t.	n.t.	18.39
18487	3628	MTN4	*A. terreus*	n.t.	n.t.	n.t.
18488	3522	MTN5	*A. terreus*	n.t.	n.t.	n.t.
18489	3523	MTN6	*A. terreus*	n.t.	n.t.	172.39
18490	3525	MTN8	*A. terreus*	n.t.	n.t.	212.65
18491	3526	MTN9	*A. terreus*	n.t.	n.t.	n.t.
18492	3527	MTN10	*A. terreus*	n.t..	n.t.	n.t.
18493	3529	MTN13	*A. terreus*	n.t.	n.t.	n.t.
18494	3546	MTN31	*A. terreus*	n.t.	n.t.	124.57
18495	3574	MTN64	*A. terreus*	n.t.	n.t.	7.88
18496	3577	MTN67	*A. terreus*	n.t.	n.t.	n.t.
18497	3578	MTN68	*A. terreus*	n.t.	n.t.	62.05
18498	3634	MTN71	*A. terreus*	n.t.	n.t.	7.16
18499	3582	MTN73	*A. terreus*	n.t.	n.t.	n.t.
18500	3588	MTN79	*A. terreus*	n.t.	n.t.	154.65
18501	3589	MTN80	*A. terreus*	n.t.	n.t.	10.70
18502	3636	MTN89	*A. terreus*	n.t.	n.t.	n.t.
18503	3601	MTN94	*A. terreus*	n.t.	n.t.	0.20
18504	3602	MTN95	*A. terreus*	n.t.	n.t.	510.56
18505	3603	MTN97	*A. terreus*	n.t.	n.t.	n.t.
18506	3639	MTN111	*A. terreus*	n.t.	n.t.	n.t.
18507	3615	MTN113	*A. terreus*	n.t.	n.t.	59.46

A. = *Aspergillus*; n.t. = not tested; * = on YES medium; LOD FB2 = 0.03 μg/g; LOD OTA = 0.015 μg/g; LOD PAT = 0.005 μg/g based on a signal to noise ratio of 3:1.

**Table 3:** GPS coordinates of the geographical origins of rhizosphere samples.

Location Name	Latitude	Longitude
Karun	32.290077	48.601034
Haft Tappeh	32.104158	48.769371
Imam Khomeini	31.816312	48.835920
Dehkhoda	31.363858	48.754828
Debal Khazaei	31.151123	48.511743
Amir Kabir	31.030506	48.278718
Salman Farsi	30.974513	48.657927
Mirza. K. Khan	30.739912	48.066038

## References

[1] FAO (Food and Agriculture Organization of the United Nations) FAOSTAT. http://faostat.fao.org/faostat/.

[2] Lozano F.J., Lozano R. (2018). Assessing the potential sustainability benefits of agricultural residues: Biomass conversion to syngas for energy generation or to chemicals production. J. Clean. Prod..

[3] Kumar D., Jain V.K., Shanker G., Srivastava A. (2003). Citric acid production by solid state fermentation using sugarcane bagasse. Process Biochem..

[4] Varga J., Kevei F., Hamari Z., Toth B., Teren J., Croft H., Kozakiewicz Z., Samson R.A., Pitt J.I. (2000). Genotypic and phenotypic variability among black aspergilli. Integration of Modern Taxonomic Methods for Penicillium and Aspergillus Classification.

[5] Abarca M.L., Accensi F., Cano J., Cabañes F.J. (2004). Taxonomy and significance of black aspergilli. Antonie Van Leeuwenhoek.

[6] Valero A., Oliván A., Marín S., Sancis V., Ramos A.J. (2007). Effect of intra and interspecific interaction on OTA production by *A.* section *Nigri* in grapes during dehydration. Food Microbiol..

[7] Chávez R., Bull P., Eyzaguirre J. (2006). The xylanolytic enzyme system from the genus *Penicillium*. J. Biotechnol..

[8] Ichishima E. (2016). Development of enzyme technology for *Aspergillus oryzae*, *A. sojae*, and *A. luchuensis*, the national microorganisms of Japan. Biosci. Biotechnol. Biochem..

[9] Samson R.A., Visagie C.M., Houbraken J., Hong S.B., Hubka V., Klaassen C.H.W., Perrone G., Seifert K.A., Susca A., Tanney J.B. (2014). Phylogeny, identification and nomenclature of the genus *Aspergillus*. Stud. Mycol..

[10] Zambrano A.Y., Demey J.R., Fuchs M., González V., Rea R., De Sousa O., Gutiérrez Z. (2003). Selection of sugarcane plants resistant to SCMV. Plant. Sci..

[11] Abdallah M., Krska R., Sulyok M. (2016). Mycotoxin contamination in sugarcane grass and juice: First report on detection of multiple mycotoxins and exposure assessment for aflatoxins B1 and G1 in humans. Toxins.

[12] Graham M.H., Haynes R.J. (2006). Organic matter status and the size, activity and metabolic diversity of the soil microbial community in the row and inter-row of sugarcane under burning and trash retention. Soil Biol. Biochem..

[13] De Souza R.S., Okura V.K., Armanhi J.S., Jorrín B., Lozano N., da Silva M.J., González-Guerrero M., de Araújo L.M., Verza N.C., Bagheri H.C. (2016). Unlocking the bacterial, and fungal communities assemblages of sugarcane microbiome. Sci. Rep..

[14] Kamei K., Watanabe A. (2005). *Aspergillus* mycotoxins and their effect on the host. Med. Mycol..

[15] Herbrecht R., Letscher-Bru V., Oprea C., Lioure B., Waller J., Campos F., Villard O., Liu K.-L., Natarajan-Amé S., Lutz P. (2002). *Aspergillus* galactomannan detection in the diagnosis of invasive aspergillosis in cancer patients. J. Clin. Oncol..

[16] Schuster E., Dunn-Coleman N., Frisvad J.C., Van Dijck P.W. (2002). On the safety of *Aspergillus niger*—A review. Appl. Microbiol. Biotechnol..

[17] Daiani M.S., Batista L.R., Rezende E.F., Fungaro M.H.P., Sartori D., Alves E. (2011). Identification of fungi of the genus *Aspergillus* section *Nigri* using polyphasic taxonomy. Braz. J. Microbiol..

[18] Nielsen K.F., Mogensen J.M., Johansen M., Larsen T.O., Frisvad J.C. (2009). Review of secondary metabolites and mycotoxins from the *Aspergillus niger* group. Annal. Bioanal. Chem..

[19] Jahromi M.F., Liang J.B., Ho Y.W., Mohamad R., Goh Y.M., Shokryazdan P. (2012). Lovastatin production by *Aspergillus terreus* using agro-biomass as substrate in solid state fermentation. J. Biomed. Biotechnol..

[20] Bizukojc M., Pawlak M., Boruta T., Gonciarz J. (2012). Effect of pH on biosynthesis of lovastatin and other secondary metabolites by *Aspergillus terreus* ATCC 20542. J. Biotechnol..

[21] Samson R.A., Peterson S.W., Frisvad J.C., Varga J. (2011). New species in *Aspergillus* section *Terrei*. Stud. Mycol..

[22] Luque M.I., Rodríguez A., Andrade M.J., Gordillo R., Rodríguez M., Córdoba J.J. (2011). Development of a PCR protocol to detect patulin producing moulds in food products. Food Control.

[23] Moake M.M., Padilla-Zakour O.I., Worobo R.W. (2005). Comprehensive review of patulin control methods in foods. Compr. Rev. Food Sci. Food Saf..

[24] Baumgardner D.J. (2012). Soil-related bacterial and fungal infections. J. Am. Board Fam. Med..

[25] Berg G., Eberl L., Hartmann A. (2005). The rhizosphere as a reservoir for opportunistic human pathogenic bacteria. Environ. Microbiol..

[26] Leger R.J.S., Screen S.E., Shams-Pirzadeh B. (2000). Lack of host specialization in *Aspergillus flavus*. Appl. Environ. Microbiol..

[27] Klich M.A. (2002). Identification of Common Aspergillus Species.

[28] Hariprasad P., Vipin A.V., Karuna S., Raksha R.K., Venkateswaran G. (2015). Natural aflatoxin uptake by sugarcane (*Saccharum officinaurum L.*) and its persistence in jaggery. Environ. Sci. Pollut. Res..

[29] Palumbo J.D., Baker J.L., Mahoney N.E. (2006). Isolation of bacterial antagonists of *Aspergillus flavus* from almonds. Microb. Ecol..

[30] Djossou O., Perraud-Gaime I., Mirleau F.L., Rodriguez-Serrano G., Karou G., Niamke S., Ouzari I., Boudabous A., Roussos S. (2011). Robusta coffee beans post-harvest microflora: *Lactobacillus plantarum* sp. as potential antagonist of *Aspergillus carbonarius*. Anaerobe.

[31] Gajera H.P., Vakharia D.N. (2010). Molecular and biochemical characterization of *Trichoderma* isolates inhibiting a phytopathogenic fungi *Aspergillus niger* Van Tieghem. Physiol. Mol. Plant. Pathol..

[32] Hill R.A., Blankenship P.D., Cole R.J., Sanders T.H. (1983). Effects of soil moisture and temperature on preharvest invasion of peanuts by the *Aspergillus flavus* group and subsequent aflatoxin development. Appl. Environ. Microbiol..

[33] Susca A., Proctor R.H., Morelli M., Haidukowski M., Gallo A., Logrieco A.F., Moretti A. (2016). Variation in fumonisin and ochratoxin production associated with differences in biosynthetic gene content in *Aspergillus niger* and *A. welwitschiae* isolates from multiple crop and geographic origins. Front. Microbiol..

[34] Medina A., Mateo R., López-Ocana L., Valle-Algarra F.M., Jiménez M. (2005). Study of Spanish grape mycobiota and ochratoxin A production by isolates of *Aspergillus tubingensis* and other members of *Aspergillus* section *Nigri*. Appl. Environ. Microbiol..

[35] Susca A., Proctor R.H., Butchko R.A., Haidukowski M., Stea G., Logrieco A.F., Moretti A. (2014). Variation in the fumonisin biosynthetic gene cluster in fumonisin-producing and nonproducing black aspergilli. Fungal Genet. Biol..

[36] Marino A., Fiorentino C., Spataro F., Nostro A. (2014). Effect of temperature on production of ochratoxin A by *Aspergillus niger* in orange juice. J. Toxins.

[37] Abarca M.L., Bragulat M.R., Castella G., Cabañes F.J. (1994). Ochratoxin A production by strains of *Aspergillus niger* var. *niger*. Appl. Environ. Microbiol..

[38] Singleton L.L., Mihailm J.D., Rush C.M. (1993). Methods for research on soilborne phytopathogenic fungi. The American Phytopathological Society.

[39] Bills G.F., Christensen M., Powell M., Thorn G., Muller G.M., Bills G.F., Foster M.S. (2004). Saprobic soil fungi. Biodiversity of Fungi: Inventory and Monitoring Methods.

[40] Johnson L.F., Curl E.A., Bond J.H., Fribourg H.A. (1960). Methods for Studying Soil Mycoflora: Plant Disease Relationships.

[41] Warcup I.M. (1960). Method for Isolation and Estimation of Activity of Fungi in Soil. The Ecology of Soil. An International Symposium.

[42] Gams W., Howekstra E.S., Aptroot A. (1998). CBS Course of Mycology.

[43] Raper K.B., Fennell D.I. (1965). The Genus Aspergillus.

[44] Pitt J., Hocking A. (2009). The ecology of Fungal Food Spoilage. Fungi and Food Spoilage.

[45] Samson R.A., Houbraken J., Thrane U., Frisvad J.C., Andersen B. (2010). Food and Indoor Fungi.

[46] Glass N.L., Donaldson G.C. (1995). Development of primer sets designed for use with the PCR to amplify conserved genes from filamentous ascomycetes. Appl. Environ. Microbiol..

[47] O’Donnell K., Nirenberg H.I., Aoki T., Cigelnik E. (2000). A Multigene phylogeny of the *Gibberellafujikuroi* species complex: Detection of additional phylogenetically distinct species. Mycoscience.

[48] Edgar R.C. (2004). MUSCLE: Multiple sequence alignment with high accuracy and high throughput. Nucleic Acids Res..

[49] Kumar S., Stecher G., Tamura K. (2016). MEGA7: Molecular evolutionary genetics analysis version 7.0 for bigger datasets. Mol. Biol. Evol..

[50] Tamura K., Nei M. (1993). Estimation of the number of nucleotide substitutions in the control region of mitochondrial DNA in humans and chimpanzees. Mol. Biol. Evol..

[51] Felsenstein J. (1985). Confidence limits on phylogenies: An approach using the bootstrap. Evolution.

[52] Association of Official Analytical Chemists (AOAC) Official Method 2008-08. http://www.aoacofficialmethod.org/index.php?main_page=product_info&cPath=1&products_id=2816.

[53] CEN (European Committee for Standardization) (2002). Foodstuffs-Determination of patulin in fruit juice and fruit based puree for young children—HPLC method with liquid/liquid partition clean-up and solid phase extraction and UV determination. CEN/TC 275/WG5. Food analysis–Biotoxins–Foodstuffs–EN 15890.

[54] Sewram V., Nair J.J., Nieuwoudt T.W., Leggott N.L., Shephard G.S. (2000). Determination of patulin in apple juice by high-performance liquid chromatography–atmospheric pressure chemical ionization mass spectrometry. J. Chromat A.

[55] Veršilovskis A., De Saeger S., Mikelsone V. (2008). Determination of sterigmatocystin in beer by high performance liquid chromatography with ultraviolet detection. W. Mycotox. J..

